# Editorial on Special Issue “Recent Developments in Food Gels”

**DOI:** 10.3390/gels9110899

**Published:** 2023-11-13

**Authors:** Kandi Sridhar, Minaxi Sharma, Baskaran Stephen Inbaraj

**Affiliations:** 1 Department of Food Technology, Karpagam Academy of Higher Education (Deemed to Be University), Coimbatore 641021, India; 2 CARAH ASBL, Rue Paul Pastur, 11-7800 Ath, Belgium; 3 Department of Food Science, Fu Jen Catholic University, New Taipei City 242062, Taiwan

Food gels have been a crucial component in the food industry for many years. They have been used to improve the texture, appearance, and stability of food products [1]. However, with recent advancements in technology and research, food gels have taken a giant leap forward, offering new possibilities and potential benefits for the global food industry [2]. While significant progress has been made in synthetic polymer chemistry to overcome some of the challenges in gel design, emerging challenges, such as sustainability, renewability, and cost-effectiveness, remain to be overcome. To address these challenges, there is a growing interest in preparing gels from natural sources with low environmental impact. Food biopolymers, such as proteins and polysaccharides are particularly promising due to their affordable, edible, biocompatible, biodegradable, and renewable properties, with a diverse range of functionalities and physical gelation characteristics [3]. Compared to synthetic gels, food gels play a critical role in modern food design by imparting desired sensory, rheological, textural, and functional properties, increasing shelf-life, reducing fat, enhancing satiety, and enabling 3D printing of complex food shapes [4]. However, further research is needed to understand the fabrication methods, gelling mechanisms, and structural/mechanical properties of food gels. It is also important to investigate how these design principles impact the rheological and tribological properties of foods to improve food quality and modify nutrient delivery, without affecting the sensory properties or altering drug/bioactive targeting within the gastrointestinal tract. Therefore, a team of researchers with expertise in food gels, novel food product development, functional foods, and extraction of bioactive compounds have organized a Special Issue entitled “Recent Developments in Food Gels” to be published in Gels (ISSN 2310-2861), an international open access journal on physical (supramolecular) and chemical gel-based materials, under the section “Gel Applications”. In this Special Issue, we delved into the latest research and innovations in the world of food gels and explored the potential they hold by collecting research and review articles dealing with but not limited to:

- Food gel fabrication with novel processing methods;
- Polymerization/crosslinking methods;
- Elucidation of molecular mechanisms;
- Innovative analytical approaches to characterization, molecular structure–functionality relationships, and food gel–body interaction.

This Special Issue presents a collection of research articles, reviews, and perspectives that highlight some of the recent advances in this rapidly evolving field. The articles in this issue cover a broad range of topics, including the use of novel gelation agents, the characterization of the structure and rheological properties of food gels, and the application of food gels in novel food products. For this Special Issue, we had invited globally renowned researchers to contribute to this topic, resulting in a total of 16 submissions, out of which 13 articles were accepted after rigorous peer-review for final publication.

One of the key themes in this issue is the addition of novel ingredients to gels and studying their characteristics. For example, Popov et al. investigated the effects of incorporating hogweed pectin into an apple pectin hydrogel. The researchers found that the addition of hogweed pectin led to an increase in the gelation temperature and gel strength of the apple pectin hydrogel. Additionally, the hogweed pectin was found to improve the sensory properties of the hydrogel, specifically its texture and overall acceptability. The study also found that the concentration of hogweed pectin had an impact on the properties of the hydrogel, with higher concentrations resulting in a stronger and more elastic gel. However, excessively high concentrations of hogweed pectin led to a decrease in the sensory properties of the hydrogel, likely due to a more pronounced earthy flavor. Similarly, another study by Arai et al. used wet-ground okra to improve the mechanical properties and intermolecular forces of soybean protein isolate (SPI) gels. The results showed that the incorporation of wet grinder-treated okra into SPI gels improved their mechanical properties, including hardness and elasticity, compared to control gels without okra. Likewise, a study investigated the effect of oxidation modification induced by malondialdehyde on the physicochemical properties and gel characteristics of duck myofibrillar proteins. The results showed that the oxidation modification caused significant changes in the physicochemical properties of the proteins, such as decreased solubility and increased surface hydrophobicity. Furthermore, the gels made from the modified proteins had lower a gel strength, water-holding capacity, and thermal stability compared to the non-modified proteins. All these studies suggested that the addition and/or modification of different ingredients can significantly affect the properties of the resulting gels, which could have implications in the food industry for the development of products with desirable textures and functional properties.

Several articles in this issue describe the enrichment of food gels with plant-based sources. For instance, Belova et al. explored the potential of using callus tissue of narrow-leaved lupin (*Lupinus angustifolius*) as a bioactive filler in 3D-printed k-carrageenan food gels. The researchers prepared the callus tissue using a tissue culture technique and characterized its chemical composition and antioxidant activity. They then incorporated the callus tissue into the k-carrageenan gel and evaluated the effects on the physical, mechanical, and rheological properties of the gel. The results showed that the incorporation of the callus tissue improved the antioxidant activity of the gel and did not significantly affect its physical and mechanical properties. With a similar approach, a study by Carvajal-Mena et al. optimized the 3D printing parameters for salmon gelatin gels using artificial neural networks and the response surface methodology. The study considered the interactions between different independent variables and used artificial neural networks to train and learn from the experimental data of the response variables. The authors performed a regression analysis of training, validation, and testing for all the experiments of apparent viscosity, hardness, and dimensional stability. The optimal printing conditions were found to be 24 mm/s for the extrusion speed, 0.70 mm for the nozzle diameter, and 0.50 mm for the nozzle height to achieve 380.85 Pa·s for viscosity, 7.75 N for hardness, and 98.87% for dimensional stability. These predicted values were not significantly different from the experimental data under the optimal printing conditions. Another study by Popov, Smirnov, Paderin, Khramova. et al. used the antioxidant pectin obtained from fireweed (*Chamaenerion angustifolium*) to improve the mechanical and rheological properties of an agar gel. This study further evaluated the effect of adding different concentrations of pectin on the mechanical and rheological properties of the agar gel, as well as its simulated digestibility and oral processing. The results showed that the addition of pectin increased the gel strength, elasticity, and viscosity, and improved its antioxidant properties. The simulated digestion study revealed that the pectin-enriched gel had a slower rate of disintegration, indicating improved stability and the potential for controlled release of bioactive compounds. The sensory analysis showed that the gel with the highest concentration of pectin had the highest overall acceptance. Therefore, these studies highlighted the potential of incorporating plant-based materials for the development of functional gels with improved mechanical, rheological, and antioxidant properties.

Food gels can play an important role in coating applications to provide a smooth and uniform coating, which can help to prevent moisture loss and oxidation, and protect the food product from contamination. With the aim of developing a composite edible coating, Heristika et al. used gelatin–pectin incorporating garlic essential oil on the physicochemical characteristics of red chili (*Capsicum annuum* L.). The study found that the edible coating improved the shelf life of the red chili peppers by reducing the rate of moisture loss and weight loss. Additionally, the coating was found to have antimicrobial properties, which inhibited the growth of bacteria on the surface of the peppers. Another study by Trodtfeld et al. developed a composite gel made from biodegradable compounds, including prolamin, d-mannose, and citric acid, as a coating to increase the oxygen barrier of food packaging materials. The gels were physically cross-linked with particles synthesized from tetraethyl orthosilicate and tetramethyl orthosilicate precursors to improve stability and the mechanical properties. The article concluded that the composite gel holds promise for oxygen-barrier food packaging and is safe for consumer contact, but further research is needed to optimize the stability of the oxygen barrier in humid environments and investigate the potential sensitizing effects of biodegradable materials on consumers. These findings have implications for the development of new food preservation technologies that incorporate natural ingredients such as garlic essential oil.

Similarly, Fauzan et al. developed an eco-friendly, biodegradable, and sustainable active packaging material using fish gelatin-based edible film incorporated with *Ficus carica* L. leaf extract. The results showed that adding *Ficus carica* L. leaf extract to gelatin films significantly affected their tensile strength, elongation at break, transmittance and transparency, solubility, water vapor permeability, antioxidant activities, and antibacterial activity. The most promising result was obtained in the edible film with 10% *Ficus carica* L. leaf extract among all the samples. The study’s overall findings showed that the fish gelatin-based films incorporating *Ficus carica* L. leaf extract have a good potential as an eco-friendly, biodegradable, and sustainable active packaging.

Finally, this issue contains four systematic review articles that explore the principles and formation mechanisms of hydrogels, and their current status and various applications in the food industry. Nath et al. summarize the various applications of food hydrogels, such as in food texture modification, nutrient delivery, and 3D printing, thereby highlighted the potential of hydrogels as a versatile tool for designing innovative food products. Another review by Kaur et al. provides an overview of the current state of research on milk protein-based nanohydrogels. The authors discuss the various methods for synthesizing milk protein-based nanohydrogels, their unique properties such as biocompatibility, stability, ability to encapsulate bioactive compounds, and potential applications of milk protein-based nanohydrogels in the food industry. Similarly, another article discusses the formulation, processing, and potential applications of food emulsion gels made from plant-based ingredients. The review concludes that the emulsion gels were semi-solid systems with a gel network structure that integrate the characteristics of emulsions and gels. These are used in the food industry to create texture, deliver functional food ingredients, and reduce the fat content of products. The article also discusses that plant-based emulsion gels have promising prospects as a delivery system for functional ingredients and as a healthy alternative to traditional fats. A review by Said et al. discusses the extraction and characterization of pectin from various sources, as well as the preparation of pectin hydrogels using different methods. The review focuses on the various crosslinking methods used to form hydrogels, with a focus on physical, chemical, and interpenetrating polymer network approaches. The article also highlights the potential applications of pectin hydrogels in the food industry, such as encapsulating bioactive substances, improving stability, and controlled release.

In conclusion, recent advances in food gels have been nothing short of revolutionary for the food industry. From the development of plant-based gels to the increased use of natural gelling agents, these innovations are changing the way we think about food and the role gels play in preserving and improving the quality of food products. These developments are not only improving the sustainability of the food industry, but also providing new opportunities for food product innovation and the creation of healthier, more functional food products. The future of food gels is exciting and holds even more potential for the food industry. We encourage researchers and companies to continue exploring the possibilities of food gels and to push the boundaries of what is possible. We believe that with continued research and innovation, food gels will play an even greater role in shaping the food industry in the years to come. We hope that this issue will provide valuable insights and inspiration for those in the food industry and spark further discussion and research in this field.

## List of Contributions

1. Popov, S.; Smirnov, V.; Khramova, D.; Paderin, N.; Chistiakova, E.; Ptashkin, D.; Vityazev, F. Effect of hogweed pectin on rheological, mechanical, and sensory properties of apple pectin hydrogel. *Gels* **2023**, *9*, 225.
2. Arai, Y.; Nishinari, K.; Nagano, T. Wet grinder-treated okara improved both mechanical properties and intermolecular forces of soybean protein isolate gels. *Gels* **2022**, *8*, 616.
3. Zhu, X.; Ma, Z.; Zhang, X.; Huang, X.; Liu, J.; Zhuang, X. Effect of malondialdehyde-induced oxidation modification on physicochemical changes and gel characteristics of duck myofibrillar proteins. *Gels* **2022**, *8*, 633.
4. Belova, K.; Dushina, E.; Popov, S.; Zlobin, A.; Martinson, E.; Vityazev, F.; Litvinets, S. Enrichment of 3D-printed k-carrageenan food gel with callus tissue of narrow-leaved lupin *Lupinus angustifolius*. *Gels* **2023**, *9*, 45.
5. Carvajal-Mena, N.; Tabilo-Munizaga, G.; Saldaña, M.D.A.; Pérez-Won, M.; Herrera-Lavados, C.; Lemus-Mondaca, R.; Moreno-Osorio, L. Three-dimensional printing parameter optimization for salmon gelatin gels using artificial neural networks and response surface methodology: Influence on physicochemical and digestibility properties. *Gels* **2023**, *9*, 766.
6. Popov, S.; Smirnov, V.; Paderin, N.; Khramova, D.; Chistiakova, E.; Vityazev, F.; Golovchenko, V. Enrichment of agar gel with antioxidant pectin from fireweed: Mechanical and rheological properties, simulated digestibility, and oral processing. *Gels* **2022**, *8*, 708.
7. Heristika, W.; Ningrum, A.; Supriyadi; Munawaroh, H.S.H.; Show, P.L. Development of composite edible coating from gelatin-pectin incorporated garlic essential oil on physicochemical characteristics of red chili *(Capsicum annnum* L.). *Gels* **2023**, *9*, 49.
8. Trodtfeld, F.; Tölke, T.; Wiegand, C. Developing a prolamin-based gel for food packaging: In-vitro assessment of cytocompatibility. *Gels* **2023**, *9*, 740.
9. Fauzan, H.R.; Ningrum, A.; Supriyadi, S. Evaluation of a fish gelatin-based edible film incorporated with *Ficus carica* L. leaves extract as active packing. *Gels* **2023**, *9*, 1–13.
10. Nath, P.C.; Debnath, S.; Sridhar, K.; Inbaraj, B.S.; Nayak, P.K.; Sharma, M. A comprehensive review of food hydrogels: Principles, formation mechanisms, microstructure, and its applications. *Gels* **2023**, *9*, 1.
11. Kaur, M.; Bains, A.; Chawla, P.; Yadav, R.; Kumar, A.; Inbaraj, B.S.; Sridhar, K.; Sharma, M. Milk protein-based nanohydrogels: Current status and applications. *Gels* **2022**, *8*, 432.
12. Yiu, C.C.-Y.; Liang, S.W.; Mukhtar, K.; Kim, W.; Wang, Y.; Selomulya, C. Food emulsion gels from plant-based ingredients: Formulation, processing, and potential applications. *Gels* **2023**, *9*, 366.
13. Said, N.S.; Olawuyi, I.F.; Lee, W.Y. Pectin hydrogels: Gel-Forming behaviors, mechanisms, and food applications. *Gels* **2023**, *9*, 732.
